# The Effect of Zr Addition on Melting Temperature, Microstructure, Recrystallization and Mechanical Properties of a Cantor High Entropy Alloy

**DOI:** 10.3390/ma14205994

**Published:** 2021-10-12

**Authors:** Enrico Gianfranco Campari, Angelo Casagrande, Elena Colombini, Magdalena Lassinantti Gualtieri, Paolo Veronesi

**Affiliations:** 1 Department of Physics and Astronomy, Alma Mater Studiorum-University of Bologna, Viale Berti Pichat 6/2, 40127 Bologna, Italy; 2 Department of Industrial Engineering, Alma Mater Studiorum-University of Bologna, Viale Risorgimento 4, 40136 Bologna, Italy; angelo.casagrande@unibo.it; 3 Department of Engineering “Enzo Ferrari”, University of Modena and Reggio Emilia, Via Pietro Vivarelli 10, 41125 Modena, Italy; elena.colombini@unimore.it (E.C.); magdalena.gualtieri@unimore.it (M.L.G.); paolo.veronesi@unimore.it (P.V.)

**Keywords:** high entropy alloy, microstructure, vacuum induction melting, heat treatment, mechanical spectroscopy, zirconium

## Abstract

The effect of Zr addition on the melting temperature of the CoCrFeMnNi High Entropy Alloy (HEA), known as the “Cantor’s Alloy”, is investigated, together with its micro-structure, mechanical properties and thermomechanical recrystallization process. The base and Zr-modified alloys are obtained by vacuum induction melting of mechanically pre-alloyed powders. Raw materials are then cold rolled and annealed. recrystallization occurred during the heat treatment of the cold-rolled HEA. The alloys are characterized by X-ray diffraction, electron microscopy, thermal analyses, mechanical spectroscopy and indentation measures. The main advantages of Zr addition are: (1) a fast vacuum induction melting process; (2) the lower melting temperature, due to Zr eutectics formation with all the Cantor’s alloy elements; (3) the good chemical alloy homogeneity; and (4) the mechanical properties improvement of re-crystallized grains with a coherent structure. The crystallographic lattice of both alloys results in FCC. The Zr-modified HEA presents a higher recrystallization temperature and smaller grain size after recrystallization with respect to the Cantor’s alloy, with precipitation of a coherent second phase, which enhances the alloy hardness and strength.

## 1. Introduction

The first scientific papers on High Entropy Alloys (HEAs) were published in 2004 [1]. Since then, the unique properties of these materials have attracted attention from research groups all over the world. In the pioneering studies by Cantor et al., it was shown that a single-phase solid solution could be obtained using equimolar contents of Cr, Mn, Fe, Co and Ni [1]. This rather un-expected result paved the way for the development of a new class of alloys [1,2,3].

HEAs may contain main elements, each with a concentration in the 5–35 at. % range [2]. The alloys present a high mixing entropy in their liquid state [3] and thus give rise to a solid solution (mostly with FCC or BCC lattice) rather than inter-metallic phases. Some promising technological features of HEAs are high hardness [4], good wear resistance [5], excellent strength at both high and low temperatures [6,7,8] and a good resistance to oxidation and corrosion [9]. The unique properties of the HEAs are ascribed to the inherent properties of a multicomponent solid solution, such as a distorted lattice structure [10], cocktail effect [11], sluggish diffusion [4] and nano-scale twinning [8].

There are four main approaches to prepare HEAs: (1) from the liquid state (arc or induction melting), (2) from the solid state (mechanical alloying, powder metallurgy), (3) from the gas state (sputtering techniques, mainly for coatings) and (4) from electrochemical processes (again, mainly for coatings) [12,13]. If we consider the production process from the liquid state, induction melting offers several advantages over arc melting such as faster chemical homogenization and preservation of low-melting-point elements. In addition, arc melting requires several re-melting steps to obtain a good homogeneity. As a result, elements with high vapor tension, such as Mn, tend to evaporate during the process [14,15]. Instead, the induction melting process results in good homogenization, thanks to the magnetic stirring, as well as providing a limited evaporation of low-melting-point elements [16].

A major research area, aimed at widening the potential applications of HEAs, concerns their response to thermomechanical processing [17]. The production process comprises heavy deformation and annealing with micro-structural modifications and textures development [18,19,20,21,22,23,24,25,26,27]. In recent works, the de-formation and annealing behaviour of the classical equimolar Cantor’s alloy was thoroughly investigated, showing that proper thermomechanical treatments could enhance its mechanical properties [20]. In particular, research showed that the micro-hardness, grain size and twin density depend on cold working and annealing temperature [21]. Studies on recrystallization behaviour of the CoCrFeMnNi alloy have demonstrated that a single FCC structure is obtained after rapid annealing at low temperature [28] and that successive cold rolling and annealing up to the recrystallization temperature can restore both ductility and workability [29,30,31]. The material thus obtained is useful for structural applications. According to literature data, the improved toughness of CoCrFeMnNi HEAs is explained as a result of a strong distortion of the FCC lattice due to overshooting solution as well as a reinforcement effect caused by precipitation [32]. Moreover, recent research on carbon containing CoCrFeMnNi HEAs revealed that thermomechanical treatments promoted the recrystallization of a starting dendritic structure into an equiaxed polycrystalline one [33]. 

In recent years, much work has been focused on the development of new HEA compositions with good mechanical performances [34]. A promising route is, for instance, to add Zr [35]. In fact, its addition has already been used successfully to strengthen both light alloys [36] and steels [37]. The expected strengthening mechanism related to Zr modifications of HEAs is dislocation pinning, which can be due to severe lattice distortion. This distortion can in turn be due to substitutional defects, vacancies or phase mismatch [38]. Based on previous studies on Mn-free CoCrFeNiZr_x_ (0.1 ≤ x ≤ 0.5) [35], Zr is expected to bring about recrystallization after cold de-formation and to originate a fine-grained micro-structure.

Owing due to these considerations, in this work both, equimolar and Zr-added Cantor’s alloys were produced with an alternative melting process, starting from pre-alloyed powders of CrNiFeCoMn mixed by mechanical alloying. When Zr metallic powders were added, eutectics were produced during high-temperature annealing. The resulting HEA has a lower melting temperature with respect to that of the standard procedure and an FCC crystalline structure. The main advantages of this approach with respect to the traditional techniques are the reduced time for the vacuum induction melting (VIM) process due to eutectic formation between Zr and all elements used in the Cantor’s alloy and the satisfactory chemical homogeneity obtained in a shorter time thanks to the use of pre-alloyed powders. A measure of 5 at. % Zr was added, resulting in a liquid phase formation at about 1470 K [39,40,41], to be compared with the 2500 K of a traditional arc melting process. As reported for other alloys, like AISI 304 stainless steels, the Fe phase below 15 wt. % Zr is a mixture of α-Fe and γ-Fe and becomes α-Fe (BCC) at or above 15 wt. % Zr [42]. As far as the base Cantor’s alloy is FCC, lowering the Zr content with respect to 15 wt. % is therefore expected to avoid the appearance of other crystalline structures.

The abovementioned process, with Zr addition, produced a dual-phase HEA. The majority phase resulted in an equimolar FCC Cantor alloy. The minority phase is an alloy with a different chemical composition but the same crystallographic structure. This phase nucleates between the dendrites of the matrix and increases the yield strength and hardness of the alloy compared with the base alloy. Nevertheless, since Zr addition mainly induced the formation of a coherent second phase, only a limited ductility decrease was observed, and it was possible to laminate the Zr-added alloy to the same degree of the base alloy without significant embrittlement and cracks.

The ability of a mixture to form a solid solution is calculated from the mixing enthalpy (H_mix_) and the atomic size mismatch (δ), according to the Hume Rothery rules [13]. In order to apply these concepts to the prediction of solid solution formation in the HEAs of this study, composition-weighted terms for differences in atom radii (δ_r_), electronegativity (χ) and average valence electron concentration (VEC) have been computed [43]. The balance between entropic and enthalpic contributions to the formation of a solid solution is expressed by the parameter Ω [43]:

(1) $$\Omega =\frac{T_{m}\Delta S_{\mathrm{mix}}}{\Delta H_{\mathrm{mix}}}$$

where ΔS_mix_ is the entropy of mixing and T_m_ the melting temperature. The most critical factor that determines whether an alloy crystallizes into a BCC or FCC structure is the valence electron concentration (VEC), which is calculated according to Equation (2) [44]:

(2) $$\mathrm{VEC}=\sum_{i=1}^{N}x_{i}{\mathrm{VEC}}_{i}$$

where x_i_ is the concentration of the i-th element.

A VEC value larger than 8 promotes the formation of an FCC phase. A value smaller than 6.87 a BCC phase. Co-existence of FCC and BCC phase is observed for VEC values between 6.87 and 8 [45,46]. A value up to 5 at. % Zr addition to the base Cantor’s alloy does not significantly alter the expected values of the parameters in Equations (1) and (2). Consequently, this modifying percentage was used in this work.

The limited solubility of Zr in each of the HEAs elements (minimum 0.2 at. % in α Co and maximum 2.7 at. % in α Ni) [46] and generally in FCC solid solutions, e.g., AISI 304 or 316L steels [45], promotes both precipitation phenomena and strong lattice distortions. If the precipitated phase is an FCC, like the main phase, then the mechanical behaviour of the Zr-added alloy will be similar to that of the standard Cantor’s alloy because coherent phase boundaries have negligible influence on dislocation glide. Conversely, incoherent phase boundaries yield significant impediment to dislocation glide, providing strength at the expense of ductility.

In this work, we show how this approach yields alloys with mechanical properties comparable with those of a standard alloy. Moreover, the micro-structure and recrystallization processes of the Co_20_Cr_20_Fe_20_Mn_20_Ni_20_ and Co_19_Cr_19_Ni_19_Fe_19_Mn_19_Zr_5_ are described.

## 2. Materials and Methods

Table 1 lists purity, particle size and unit cell structure of the elemental powders, provided by Sigma–Aldrich (Darmstadt, Germany), used as reactants for the HEAs preparation.

Powder mixtures were subjected to pre-alloying by mechanical milling in Argon atmosphere using a Planetary Ball Mill (PM 100 by Retsch GmbH, steel balls with BPR 15:1, Haan, Germany) working at 400 rpm. Treatment cycles of 15 min followed by 5 min break time (to avoid overheating) were performed for a total milling time of 45 h [13]. The alloyed powders, with composition Co_20_Cr_20_Fe_20_Mn_20_Ni_20_ and Co_19_Cr_19_Fe_19_Mn_19_Ni_19_Zr_5_, were placed in alumina crucibles and thermally treated in a vacuum induction furnace at temperatures exceeding 1720 K for 30 min, during which complete melting was achieved. Disks with 35 mm diameter and 8 mm height were produced. They weighted between 56 g and 58 g. Induction melting of mechanically pre-alloyed powders was used as a synthesis approach in order to reduce element segregation and Mn loss.

Sections of the as-cast disks were cold rolled using a laboratory rolling equipment to achieve a 90% thickness reduction (from 3 mm to 0.3 mm). The specimens were subsequently re-crystallized by a 30 min annealing in a Kanthal Super HT rapid high-temperature furnace (Hallstahammar, Sweden). The crystallization temperatures were determined by differential scanning calorimetry (DSC) performed in purified Helium atmosphere using a NETZSCH STA 429 CD instrument. The specimens were scanned in alumina crucibles from room temperature to 1700 K, at a heating rate of 10 K/min. DSC data were presented in a previous communication [41]. All specimens were annealed at the recrystallization temperature of the Zr-modified alloy, 1143 K, which is higher than the recrystallization temperature of the base alloy (T = 1043 K).

As-cast, cold-worked and re-crystallized specimens were analysed as follows:

- X-ray Diffraction (XRD), used to determine the crystal structure. A Θ/2Θ scan was performed in the 2Θ range from 35° to 100° using a Panalytical X’Pert PRO diffractometer equipped with a gas proportional detector (Malvern, UK). A parallel beam configuration was applied, including an X-ray mirror (incident beam optics) coupled with a long soller slit and a flat monochromator (diffracted beam optics). Hence, sample displacement errors were avoided, and a correct determination of the unit cell from peak positions could be performed.
- Micro-structural investigations were performed by optical microscopy (OM) and scanning electron microscopy in conjunction with energy dispersive spectroscopy (SEM–EDS, ZEISS EVO 50 VP, Jena, Germany). Specimens used for these analyses were polished and chemically etched (glyceregia solution composed of 1 HNO_3_ + 3 HCl + 3 Glycerol—OM Olympus GX71, Tokyo, Japan). Transmission electron microscopy analyses (TEM, JEOL JEM-2100 apparatus at accelerating voltage of 200 kV, Boston, USA) were performed on selected samples prepared by conventional twin jet electropolishing of mechanically pre-thinned (100 µm) foils. The electropolishing procedure was performed in a mixture of 95% C_2_H_5_OH and 5% HClO_4_ at a potential of 27 V. Selected area electron diffraction patterns (SAED) were collected and indexed using a known camera length calibrated using an aluminium standard. Data were elaborated using a Gatan Digital Micro-graph software (version 3.4.3, Pleasanton, CA, USA).
- Micro-hardness was measured by instrumented indentation (CSM Instruments, Ostfildern-Scharnhausen, Germany). To perform depth-sensing nano-indentation tests, two different loads were applied: 300 mN force with a linear loading/un-loading rate of 450 mN/min and 50 mN force with linear loading/un-loading rate of 150 mN/min, applied for 15 s. The indentations were performed using a Berkovich tip, and the equivalent Vickers hardness was calculated according to the Oliver and Pharr method [47]. Ten micro-hardness measurements were performed on both alloys in as-melted, work-hardened and re-crystallized states, and the average values were reported. The measurements performed on the Zr-rich alloy may have a contribution from the Zr-rich phase. Five micro-hardness measurements were performed both on the dendritic and on the inter-dendritic structure in the as melted, work-hardened and re-crystallized state of the HEA, and the average values were reported.
- Mechanical measurements were performed in a vacuum by means of the mechanical analyser VRA 1604 [48,49]. In the VRA apparatus, specimens are mounted in free-clamped mode and excited by flexural vibrations. Specimens are kept into resonance while temperature changes at the selected rate. The resonance frequency of all specimens is in the 400 to 900 Hz range; the strain amplitude is about 10^−5^. Specimens are heated from room temperature up to a maximum temperature of 700 K at 1.5 K/min rate. The internal friction (usually referred to as IF or Q^−1^) and the dynamic modulus are determined from the envelope of the decreasing oscillation amplitude of the specimen when excitation is turned off and from the specimen resonance frequency.

## 3. Results and Discussion

### 3.1. Micro-Structure Characterization

**Figure 1:**
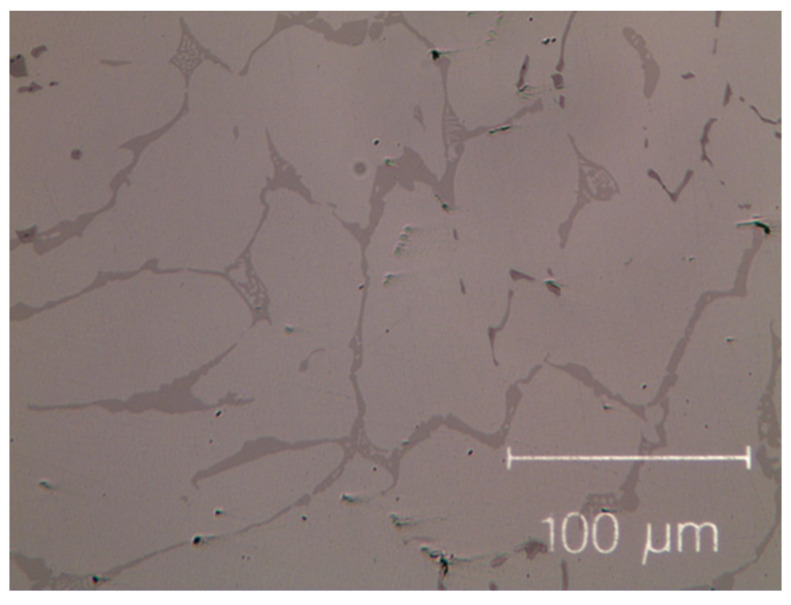
Micro-graph of as-cast Co_19_Cr_19_Ni_19_Fe_19_Mn_19_Zr_5_. Black dots, mainly dispersed in the inter-dendritic phase, are the result of contamination during the milling process.

**Figure 2:**
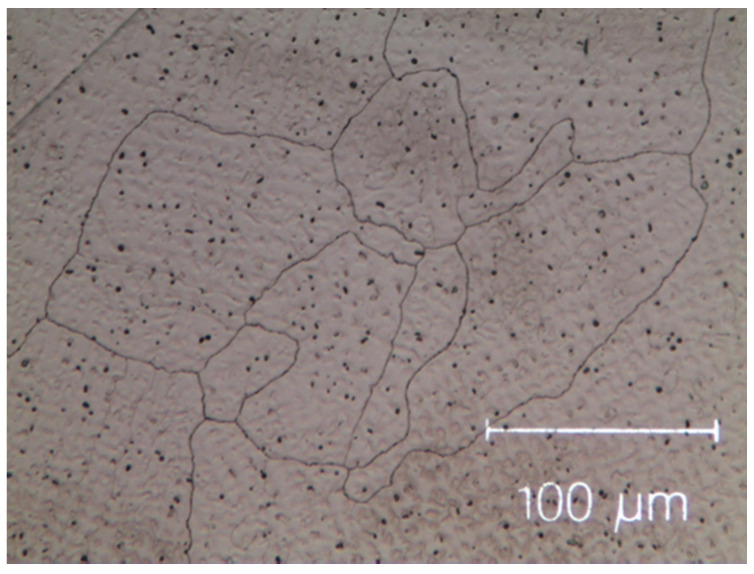
Micro-graph of as-cast Cantor’s alloy Co_20_Cr_20_Ni_20_Fe_20_Mn_20_. Black dots are the result of contamination during the milling process.

The as-cast micro-structures, shown in Figure 1 and Figure 2, provide us with representative images of the induction-melted and rapidly solidified HEAs.

The as-cast Zr-modified alloy is made of large grains. Finer dendritic grains are found in the base Cantor’s alloy, extending from the edge towards the centre of sample moulds, aligned with the heat flow direction during solidification. The raw materials are slightly contaminated during the milling process [50,51,52], mainly by particles coming from metal oxides, carbon and the wear of the zirconia balls. These contaminations are visible as black dots in Figure 1 and Figure 2. They are found in the inter-dendritic area of the Zr-free alloy and in the inter-dendritic phase in the Zr-added alloy.

Table 2 reports the results from semi-quantitative EDS analyses of the two alloys. Specifically, data refers to: (i) an extended area containing both dendritic and inter-dendritic areas and thus representative of the overall alloy chemical composition; (ii) the dendritic structure; and (iii) the inter-dendritic second phase.

**Figure 3:**
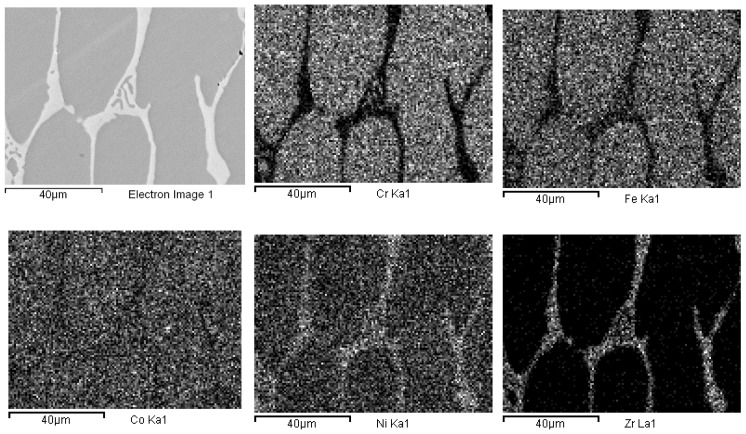

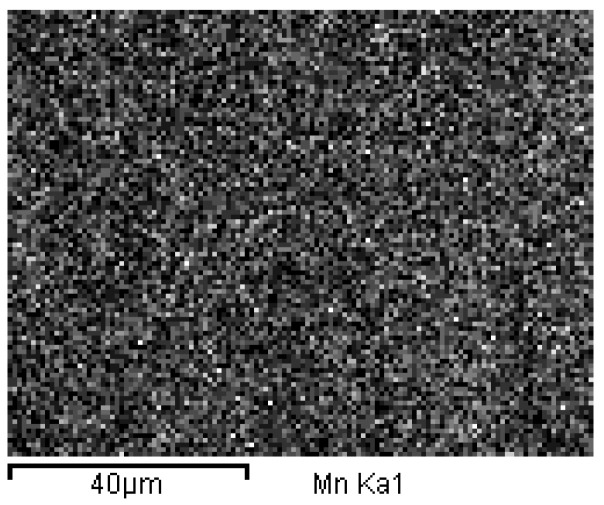
EDS mapping of as-cast Co_19_Cr_19_Ni_19_Fe_19_Mn_19_Zr_5_. The maps report, from left to right and top to bottom, the electron image of a portion of the specimen and the corresponding elemental distribution of Cr, Fe, Co, Ni, Zr, Mn.

**Figure 4:**
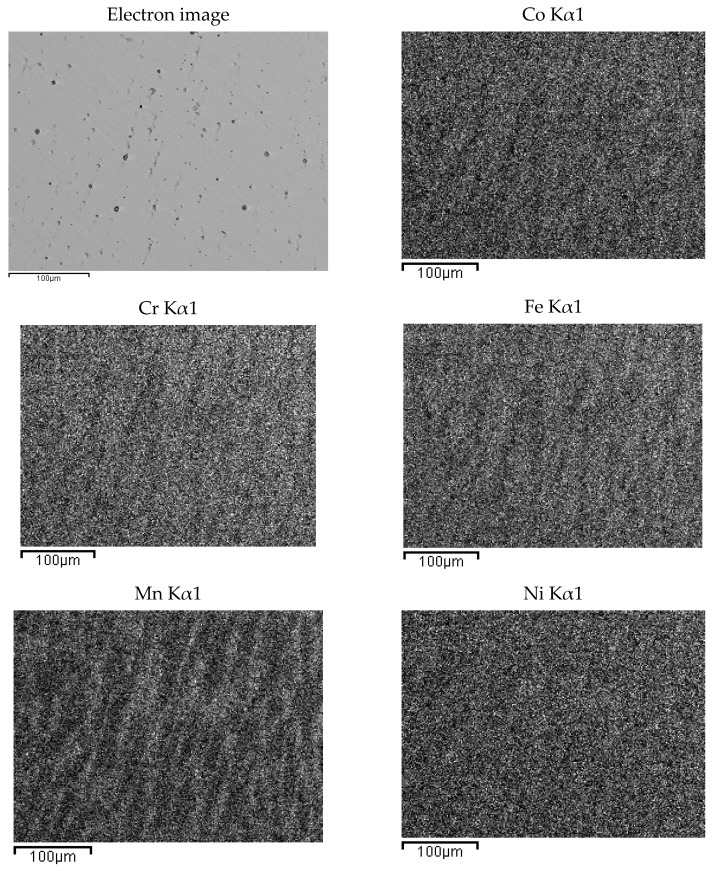
EDS mapping of as-cast Co_20_Cr_20_Ni_20_Fe_20_Mn_20_. The maps report, from left to right and top to bottom, the electron image of a portion of the specimen and the corresponding elemental distribution of Co, Cr, Fe, Mn, Ni. Kα1 transition was used for all elements.

The presence of oxygen is both due to pre-alloying and to the chemical attack, needed to highlight the alloys micro-structure. This second phase is typical of cast samples and can be clearly observed in the maps of Figure 3. In Figure 4, the same maps for the base alloy are reported for comparison.

According to EDS analyses and mapping, Co is uniformly distributed over both the dendritic and the inter-dendritic regions in the Zr-modified alloy. Dendrites are made of an equimolar solid solution of Fe, Cr, Ni and Co, the absolute amount of each constituent being slightly higher than the nominal one. Zr is mainly concentrated in the inter-dendritic regions. These areas are also Ni-enriched and Fe- and C-depleted. The inter-dendritic phase of the Zr-free alloy includes small spherical grains (8–10 µm diameter) composed of Mn and Cr oxides. The formation of such inclusions has previously been reported by several authors, and they seem difficult to be avoided in the mechanical alloying plus induction melting processing route [21]. Responsible for their presence is the pre-alloying procedure, which leads to partial powder oxidation, as demonstrated by the data reported in Table 3 and by XRD (MnO PDF card number 00-004-0326; MnCrO_4_ with PDF card number 00-033-0893, not shown here). The as-cast alloys also present deviations from the nominal composition (Table 2). Unfortunately, even though Mn volatility and the early presence of a liquid phase, due to eutectics formation [53,54,55], is reduced by induction technology compared with arc melting; nonetheless, they are still present.

**Figure 5:**
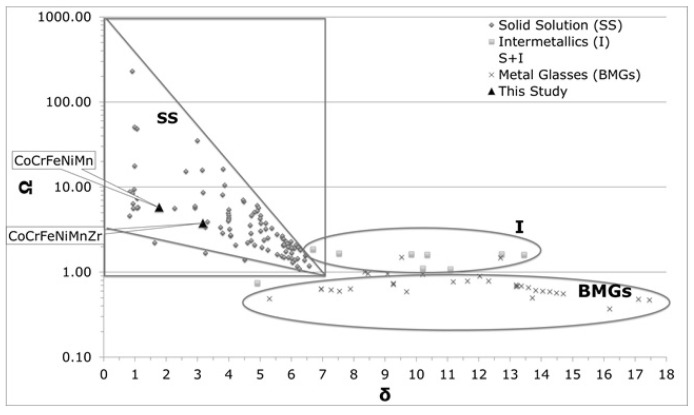
Phase formation according to Ω and δ parameters.

Table 4 reports the computed values for the Ω and δ parameters for the two alloys, together with those calculated from the compositions of the dendritic and inter-dendritic phases. In Table 5, the parameter values needed to achieve solid solutions are reported [46]. For comparative purposes, data presented in Table 4 are also plotted in Figure 5, together with values reported from the literature for other HEAs [55,56].

The parameters values obtained for Co_20_Cr_20_Fe_20_Mn_20_Ni_20_ are in agreement with those found into literature [43]. From the VEC value, an FCC structure was predicted for both systems (VEC > 7.5).

**Figure 6:**
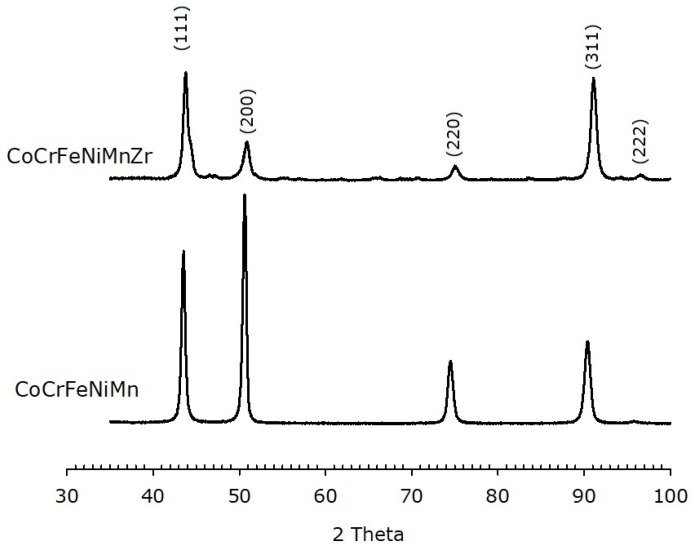
XRPD data of as-cast HEAs, with and without Zr modifier, showing an FCC structure. The Miller indices of the lattice planes are indicated.

XRD patterns of the as-cast alloys are shown in Figure 6. An FCC phase is, indeed, observed. It must be pointed out that the relative peaks intensities are different for the two alloys and different from those expected for a randomly oriented FCC metal, where the (111) peak is most intense. On the other hand, the most intense peak for the alloys here investigated corresponds to the (200) plane in the base alloy and to the (311) plane in the Zr-modified HEA, indicating a preferred orientation of the crystallites.

**Figure 7:**
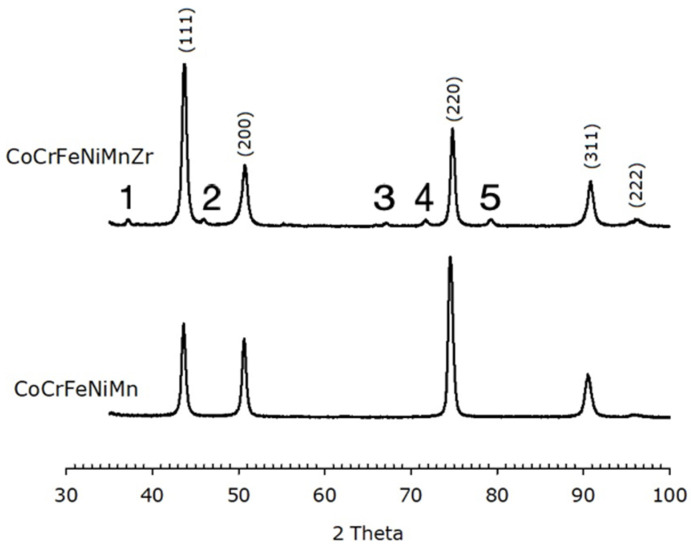
XRPD data of of Zr-CoCrFeNiMn (top) and CoCrFeNiMn (bottom) after recrystallization, showing an FCC structure. The Miller indices of the lattice planes are indicated. Peaks labelled with numbers from 1 to 5 are due to oxides. Numbers 1, 4 and 5 correspond to the (110), (121) and (220) peak of ZrO_2_, respectively. Numbers 2 and 3 are not clearly identified.

The calculated lattice parameter for the Zr-free HEA is (0.360 ± 0.002) nm, in agreement with literature [57]. The Zr-added alloy has a slightly shorter unit cell edge, i.e., (0.357 ± 0.002) nm. XRD patterns of the HEAs after annealing are reported in Figure 7. Both alloys still exhibit an FCC structure with lattice parameter (0.359 ± 0.002) nm and (0.358 ± 0.002) nm for the base and the Zr-modified alloy, respectively.

The Zr-containing alloy also exhibits low-intensity peaks associated with oxide phases. Some peaks can be indexed as ZrO_2_ peaks; the others are not clearly attributable, but EDS data suggest they are due to MnCrO_4_ and Mn_2_O_3_ (ZrO_2_, MnCrO_4_ with PDF card number 00-033-0893 and Mn_2_O_3_ with PDF card number 01-089-4836). The relative intensities of the peaks in the patterns differ from those expected from randomly oriented crystallites, like it has been found on as-cast specimens. Both annealed alloys display an intense (220) peak, which possibly indicates a <220> preferred orientation in a direction perpendicular to the recrystallization direction.

**Figure 8:**
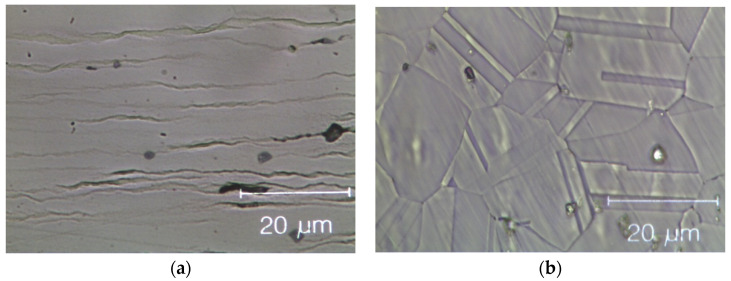

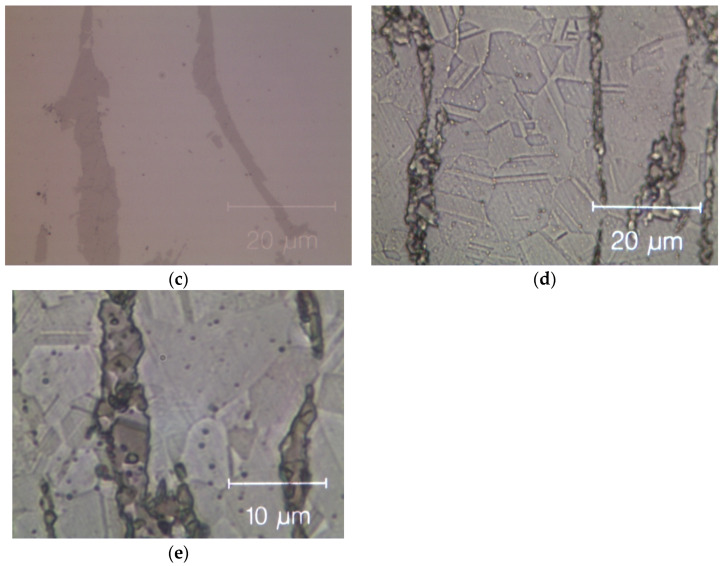
Optical microscope images of the Zr-free and Zr-modified HEA. (**a**) Zr-free after cold rolling; (**b**) Zr-free after recrystallization; (**c**) Zr-modified after cold rolling and (**d**) Zr-modified after recrystallization; (**e**) detail of Zr-modified after recrystallization: twin boundaries in the inter-dendritic phase are visible. The inter-dendritic phase is that corresponding to the dark vertical stripes in the optical image (**c**–**e**).

Figure 8 shows optical microscopy images of two HEAs after cold rolling and after recrystallization. The cold-rolled base alloy has a de-formed elongated dendritic micro-structure without cracks; see Figure 8a. After annealing, Figure 8b, twinned grains with average size (15 ± 2) µm appear (ASTM E112—13 [58]). In the Zr-added alloy, despite the presence of a Zr-rich phase in the cold-rolled specimens, cracks are still absent, as shown in Figure 8c. The fully re-crystallized micro-structure of this alloy, Figure 8d, presents finer equiaxed geminated grains with an average grain size of (6.0 ± 1.5) μm [58] in the dendritic regions and of (1.5 ± 0.5) μm into the inter-dendritic regions; see Figure 8e.

This difference in grain size between dendritic and inter-dendritic phase appears to be related to the Zr concentration. Specifically, it is the known influence of solutes atoms on recovery, recrystallization and grain growth that can help us understand the observed differences. Solute atoms can, in fact, influence the stacking fault energy and/or impede the movement and decrease the rate of dislocation climb and cross slip, which are the mechanisms underlying these processes [59].

Table 6 reports results from EDS analyses performed in the two distinct areas of the re-crystallized Zr-added alloy. When compared with as-cast data (Table 2), it is observed that the chemical composition of the dendritic area is un-changed and that Zr is still concentrated in the inter-dendritic phase. Despite Mn depletion, the Co, Cr, Fe and Ni content in the inter-dendritic phase is now close to the equi-atomic composition. The equi-atomic composition for four out of five elements encourages us to belief in that the minority phase is another FCC Cantor’s alloy with similar mechanical properties.

**Figure 9:**
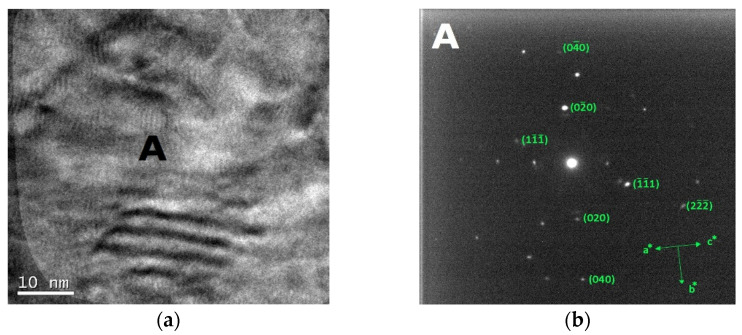
Bright field TEM images (**a**) and the corresponding SAED pattern (**b**) of the inter-dendritic phase in the CoCrFeMnNiZr alloy after cold working.

**Figure 10:**
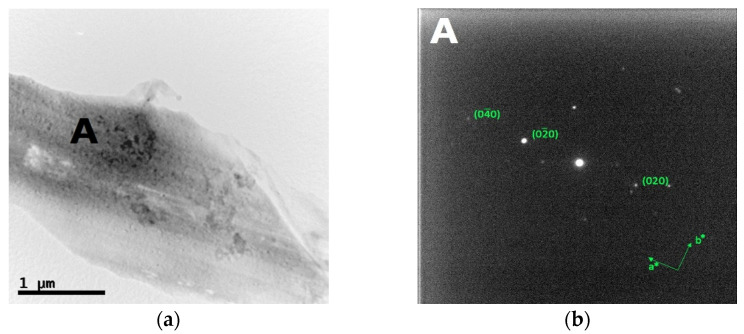
Bright field TEM images (**a**) and the corresponding SAED pattern (**b**) of the inter-dendritic phase in the CoCrFeMnNiZr alloy after recrystallization.

The bright field TEM images and the corresponding SAED patterns collected from the out of plane section of cold-worked and re-crystallized Zr-modified HEA are shown in Figure 9 and Figure 10, respectively. The images were recorded from the inter-dendritic region. The SAED patterns reveal that the inter-dendritic phase has an FCC structure (Fm3m), as expected from XRD data. The calculated lattice parameters for both the cold-worked and the re-crystallized phase are (0.36 ± 0.02) nm, in agreement with those obtained from XRD. Twin bands are visible in Figure 10, which are composed of bundles of thinner micro-twins which; due to spot size limitations, they appear as a single entity.

Available literature data indicate that only a single FCC phase exists in the CoCrFeMnNi HEA [60,61] so that all grains can be considered as belonging to the same phase. For the re-crystallized Zr-added alloy, the average grain size obtained from electron microscopy is 5.2 μm, in agreement with the (6.0 ± 1.5) μm value determined by optical microscopy.

### 3.2. Mechanical Properties

**Figure 11:**
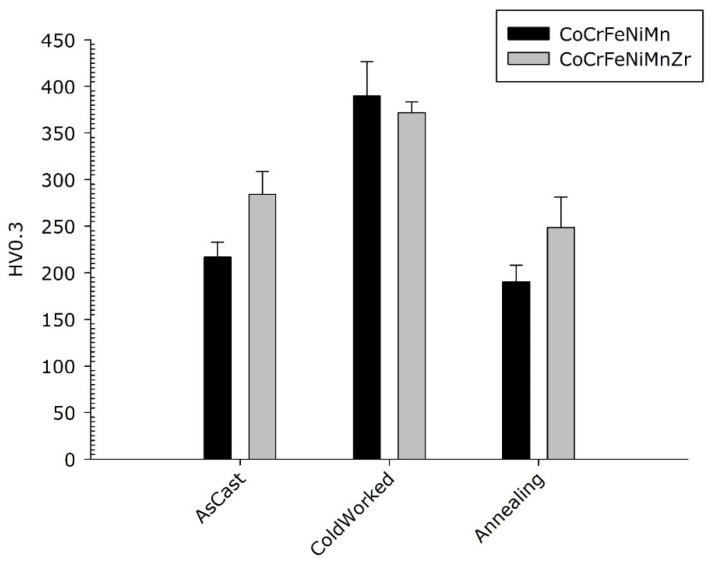
Micro-hardness of Co_20_Cr_20_Ni_20_Fe_20_Mn_20_ and Co_19_Cr_19_Ni_19_Fe_19_Mn_19_Zr_5_ alloys.

The micro-hardness of the as-cast, cold-worked and re-crystallized samples of the two HEAs compositions was measured in randomly selected locations including both dendritic and inter-dendritic phases, as described in Section 2. The results are visible in Figure 11. As reported in the optical micro-graphs of Figure 8, no cracks are visible for both compositions.

No statistically significant difference in micro-hardness is observed between the cold-worked alloys. They both harden with the typical behaviour of FCC structures with low stacking fault. As expected, as a result of the usual solute effect on hardness, the smaller hardness values were observed in the CrCoFeMnNi Cantor’s alloy in the as-cast and re-crystallized state. Noteworthy, the cold-rolled base alloy exhibits a better work hardening capability compared to the Zr-modified one.

**Figure 12:**
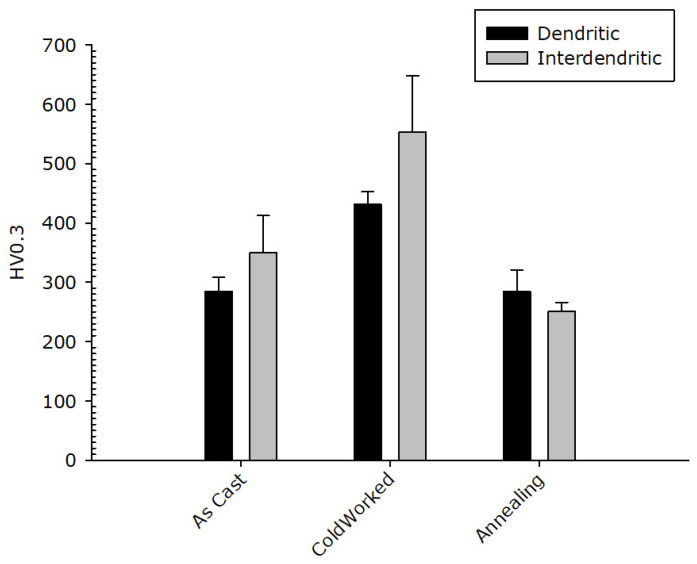
CoCrFeNiMnZr alloy: Micro-hardness value in the dendritic (black bar) and inter-dendritic (grey bar) zones.

The result of micro-hardness tests on the dendritic and the inter-dendritic phase in the CoCrFeNiMnZr alloy are reported in Figure 12. The values obtained for the Zr-rich inter-dendritic phase are higher compared to those measured in the dendritic region, in the as-cast and in the cold-rolled states, supporting the assumption of a strengthening effect for zirconium. The two phases, after recrystallization, have very similar micro-hardness values comparable with those of the as-cast alloy. A state of easy de-formation is restored so that the CoCrFeMnNiZr alloy could also be used for structural uses, highlighting the tendency to keep a ductile behaviour.

**Figure 13:**
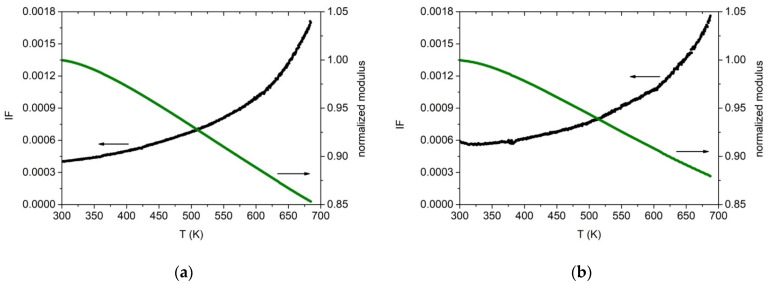
Internal friction (black) and dynamic elastic modulus (green) for cold-worked specimens with (**a**) Co_20_Cr_20_Ni_20_Fe_20_Mn_20_ and (**b**) with Co_19_Cr_19_Ni_19_Fe_19_Mn_19_Zr_5_ nominal composition. Heating rate 1.5 K/min. Resonance frequency at room temperature, 540 Hz (**a**) and 514 Hz (**b**). Modulus values are normalized to their value at 300 K.

The dynamic Young’s modulus E and damping were measured for the two alloys as explained in Section 2. Cold-rolled specimens were heated the first time in a vacuum up to 700 K in order to relax, at least partially, the stress induced by the de-formation process. Then a second thermal run was performed up to 700 K. In Figure 13 are reported the data acquired during heating at 1.5 K/min. The results closely resemble those obtained on the same Cantor’s alloy by other experimental groups and therefore prove how the induction technique used in this work is able to produce good quality material. The dynamic modulus decreases monotonically while the damping increases with the typical trend due to dislocation bending. A damping value of about 4 × 10^−4^ is measured at room temperature, which increases to 18 × 10^−4^ at 700 K. E = (140 ± 10) GPa at 300 K. The Zr-added alloy exhibits the same overall behaviour, except for a higher damping at room temperature, Q^−1^ = 6 × 10^−4^, and a lower modulus value at 300 K, E = (130 ± 10) GPa. In both cases, the uncertainty on E is mainly due to the un-even thickness of the cold-rolled specimens. These modulus values are lower than those usually obtained by computer calculations (E~200 GPa at 300 K) [62,63,64] but in agreement with other experimental results [32]. Density was measured to be (7.3 ± 0.4) g/cm^3^ and (7.6 ± 0.4) g/cm^3^ for the base and Zr-added alloy, respectively.

From a mechanical point of view, the two HEAs alloys differ little by presenting very similar mechanical properties and behaviour. The presence of a second iso-structural phase in the Zr-added alloy does not lead to the emergence of structural problems in industrial use. Both alloys are largely de-formable and tough even at low temperatures and can be reinforced by micro-twinning and grain size reduction [1,8,11].

## 4. Conclusions

In this work, for the first time, CoCrFeMnNi and 5 at. % Zr-modified CoCrFeMnNiZr HEAs were synthesized using vacuum induction melting of mechanically pre-alloyed elemental powders. The conventional strategy to obtain recrystallization of the original structure by cold de-formation followed by annealing was used for both compositions. It resulted effective in achieving an equiaxed structure suitable for structural employ. The 5 at. % Zr addition does not modify the crystalline phase of the alloy but considerably lowers the production melting temperature, and speeds up the process. The melting temperature of the CoCrFeMnNiZr alloy was found to be lower than the Zr-free counterpart, due to eutectics formation between each single element and Zr, yielding a good elemental homogenization in short times. The as-cast alloys showed a dendritic solidification micro-structure with a clear distinction between the dendritic and the inter-dendritic phases in the case of the Zr-modified alloy. The crystallographic structure of both phases resulted in FCC, in agreement with the VEC calculations. In addition, the Zr-modified HEA exhibits a significant size reduction of FCC grains compared to the Zr-free HEA, leading to a finer micro-structure.

The preliminary tests confirm the good mechanical quality of both the base and Zr-added alloys. Zr addition to the traditional Cantor alloy therefore fulfilled the objective of speeding up the process and reducing the melting temperature of the alloy without relevant embrittlement. A more thorough investigation was not possible due to the limited amount of available material and will be performed successively.

## Figures and Tables

**Table 1:** Composition of the metal powders (BCC: body centred cubic; FCC: face centred cubic; HCP: Hexagonal close packed arrangement).

Element	Purity (%)	Particle Size (μm)	Cell
Fe	97.00	<44	BCC
Co	99.80	<2	HCP
Ni	99.70	<5	FCC
Cr	99.00	<44	BCC
Mn	99.00	<75	BCC
Zr	99.80	150	HCP

**Table 2:** Semi-quantitative analysis of average area, dendritic and inter-dendritic phase of as-cast specimens (each reported atomic composition is a 5-point averaged analysis).

Element	Co_19_Cr_19_Ni_19_Fe_19_Mn_19_Zr_5_			Co_20_Cr_20_Ni_20_Fe_20_Mn_20_		
	Average Area	Dendritic	Inter-Dendritic	Average Area	Dendritic	Inter-Dendritic
O	2.33	2.17	2.14	2.88	1.63	3.24
Co	21.22	22.63	17.27	19.67	20.75	16.80
Cr	21.18	22.91	4.39	19.98	20.80	19.33
Mn	10.53	10.08	11.87	17.96	16.34	23.66
Fe	20.99	22.79	7.88	19.83	20.92	17.21
Ni	21.52	19.36	38.47	19.68	19.55	19.76
Zr	2.22	0.07	17.98	/	/	/

**Table 3:** Semi-quantitative analysis of Co_20_Cr_20_Ni_20_Fe_20_Mn_20_ (at%) powder after ball milling, as obtained from EDS.

Element	Co_20_Cr_20_Ni_20_Fe_20_Mn_20_	
	wt. %	at. %
O	2.14	7.11
Co	19.59	17.66
Cr	20.36	19.68
Mn	19.65	18.69
Fe	19.29	19.71
Ni	18.96	17.15

**Table 4:** Calculated parameters of the studied HEAs.

Alloy	Area	δ%	Ω	ΔH_mix_ [kJ/mol]	ΔS_mix_ [J/molK]	VEC	ΔΧ%
Co_20_Cr_20_Ni_20_Fe_20_Mn_20_	NOMINAL	1.73	5.7	−4.16	13.38	8	1.47
	Real Dendrite	1.77	5.84	−4.14	13.37	8.0	1.48
	Real Inter-dendritic	1.77	5.50	−4.36	13.35	8.0	1.47
Co_19_Cr_19_Ni_19_Fe_19_Mn_19_Zr_5_	NOMINAL	3.2	3.8	−6.91	14.36	7.8	1.45
	Real Dendrite	2.18	5.82	−4.19	13.23	8.0	1.49
	Real Inter-dendritic	11.17	1.23	−19.37	13.20	7.98	1.46

**Table 5:** Suitable range to form solid solution.

Phase	ΔS_mix_	ΔH_mix_	δ
Solid Solution Phase	10 < ΔS_mix_ < 19.5	−22 < ΔH_mix_ < 7	0 < δ < 8.5
BMGs	7 < ΔS_mix_ < 14	−35 < ΔH_mix_ < −8.5	δ < 9

**Table 6:** Semi-quantitative analysis of the Zr-added alloy (atomic %) as obtained from EDS.

Region	Co	Cr	Mn	Fe	Ni	Zr	O
Dendritic	22.52	21.63	9.91	21.31	20.1	0.08	1.97
Inter-dendritic	16.02	15.51	8.24	16.59	15.48	21.82	6.34

## Data Availability

The data presented in this study are available on request from the corresponding author.
